# More problems, more money: Identifying and predicting high-cost rescue after colorectal surgery

**DOI:** 10.1016/j.sopen.2023.10.007

**Published:** 2023-10-28

**Authors:** Ira L. Leeds, Miranda S. Moore, Kurt Schultz, Joseph K. Canner, Haddon J. Pantel, Anne K. Mongiu, Vikram Reddy, Eric Schneider

**Affiliations:** Yale School of Medicine, Department of Surgery, Division of Colon & Rectal Surgery, New Haven, CT, United States

**Keywords:** Colorectal surgery, Cost analysis, Complication, Logistic regression

## Abstract

**Background:**

Successful rescue after elective surgery is associated with increased healthcare costs, but costs vary widely. Treating all rescue events the same may overlook targeted opportunities for improvement. The purpose of this study was to predict high-cost rescue after elective colorectal surgery.

**Methods:**

We identified adult patients in the National Inpatient Sample (2016–2021) who underwent elective colectomy or proctectomy. Rescued patients were defined as those who underwent additional major procedures. Three groups were stratified: 1) uneventful recovery; 2) Low-cost rescue; 3) High-cost rescue. Multivariable Poisson regression was used to identify preoperative clinical predictors of high-cost versus low-cost rescue.

**Results:**

We identified 448,590 elective surgeries, and rescued patients composed 4.8 %(21,635) of the total sample. The median increase in costs in rescued patients was $25,544(*p* < 0.001). Median total inpatient costs were $95,926 in the most expensive rescued versus $34,811 in the less expensive rescued versus $16,751 in the uneventfully discharged(*p* < 0.001). When comparing the secondary procedures between the less expensive and most expensive rescued groups, the most expensive had an increased proportion of reoperation (73.4 % versus 53.0 %,*p* < 0.001). When controlling for other factors and stratification by congestive heart failure due to an interaction effect, a reoperation was independently associated with high-cost rescue (RR with CHF = 3.29,95%CI:2.69–4.04; RR without CHF = 2.29,95%CI:1.97–2.67).

**Conclusions:**

High-cost rescue after colorectal surgery is associated with disproportionately greater healthcare utilization and reoperation. For cost-conscious care, preemptive strategies that reduce reoperation-related complications can be prioritized.

## Introduction

Complications are an unfortunate but unavoidable part of surgical practice. However, not all complications are equivalently consequential. While many complications can be quickly recognized and remedied, there are infrequent complications that can lead to further deterioration and ultimately chronic critical illness or death. In the last two decades, a new emphasis in the patient safety and surgical quality literature has emphasized the importance of this latter sequence events and the resilience of an institution to arrest this deterioration cascade as a quality metric. “Failure to rescue,” defined as mortality following a complication [1], is now commonplace in the surgical quality literature and an established healthcare quality indicator [[2], [3], [4], [5]].

While rescue has secured its place as a quality measure, little is known regarding its role in healthcare value, or quality divided by costs. Evidence from patient harm literature suggests that surgical complications are a major healthcare cost burden. Responding to adverse events is responsible for 15 % of total global healthcare expenditures; the greatest burden of healthcare associated adverse events includes many related to prolonged surgical care including healthcare-associated infections, venous thromboembolism, and pressure ulcers [6]. On average, a single complication after colorectal surgery is estimated to double the costs of surgical care [7].

With regards to the costs of rescue itself, there are few published studies. The existing literature on rescue costs is confined to single payer studies for single surgery types [8,9]. In colorectal surgery, there is no estimate for the cost of rescue, or a major complication with deterioration, in the literature. Accurate assessments of healthcare costs are essential for planning and prioritizing surgical quality initiatives as well as advancing the use of novel healthcare services research methods such as cost-effectiveness analysis [10,11]. The purpose of this study was to estimate the cost of rescue and identify predictors of high-cost rescue using nationally collected all-payer administrative data. We hypothesized that highly comorbid patients and rescue requiring invasive procedural interventions would be the greatest drivers of high-cost rescue cases. This study objective and hypothesis are important for both contextualizing contemporary healthcare costs and identifying specific drivers of high resource utilization.

## Materials & methods

The following methodology was reviewed by the Yale Institutional Review Board and deemed exempt due to nonidentifiable data in a publicly available dataset.

### Data source and target population

We used the National Inpatient Sample (NIS) from 2016 to 2019 to identify all admissions for adult patients ≥18 years old with an elective colectomy or proctectomy (ICD-10-PCS codes beginning with 0DT[*E*-P, excluding J appendiceal procedures]) occurring within the first 48 h after admission (“index surgery”); non-elective and emergent admissions were excluded based on NIS structured variables. The 48-h cutoff was used to ensure non-elective cases were excluded by a second means. Admissions with missing values for patient age, sex, LOS, vital status, or total charges were additionally excluded (641 patients omitted from cost analysis; only 96 patients omitted from non-cost clinical outcomes).

The NIS is an all-payer database of inpatient admissions to US community hospitals and is part of the Healthcare Cost and Utilization Project (HCUP), sponsored by the 10.13039/100000133Agency for Healthcare Research and Quality (AHRQ). The NIS database is built as a 20 % stratified random sample of all hospital discharges collected by participating states, which represented 97 % of the US population in 2019. Primary diagnosis, up to 34 secondary diagnoses, and up to 25 procedures from the same admission are reported using ICD-10-CM and ICD-10-PCS codes. The NIS contains approximately 7 million records each year, and the data can be weighted to provide national-level estimates [12].

### Case definition

We defined patients who required rescue as those with an index surgery followed by additional administratively coded procedures (any ICD-10-PCS code beginning with “0”) occurring in the days following the operation (each ICD-10-PCS code has its own corresponding relative date of service from admission). We argue that given the limitations of administrative data and the limited and misuse of diagnostic coding, this approach more accurately ascertains those patients with serious complications. Postoperative diagnostic codes – the alternative measure that has been used for rescue – are more likely to be missed than procedure codes given their limited impact on hospital or provider billing, in contrast to procedure codes [13,14].

On preparatory work for this study, we noted a unique relationship between costs and rescue for patients who survived to discharge versus those who died. These two relationships were sufficiently distinct that combining these two outcomes into a single analysis of rescue and costs would lead to incomplete and potentially inaccurate conclusions. Patients who died during their inpatient admission were excluded and planned to be studied separately in the future.

Clinical review by authors I.L. and K.S. excluded select minor procedures including: ICD-10-PCS codes 0DH67UZ, 0DH68UZ (noninvasive oropharyngeal feeding tube placement), codes 0D9670Z, 0D9680Z (nasogastric decompress), and ICD-10-PCS code categories (by Clinical Classification Software-Refined (CCSR) groups) [15] of “venous and arterial catheter placement”, “bladder catheterization and drainage”, or “genitourinary tract procedures, NEC.”

### Identification of reoperations

We defined our primary explanatory variable to be reoperation. Post-index day ICD-10-PCS procedure codes were grouped by CCSR categories [15], which were then reviewed by I.L. and K.S. using clinical judgement to identify those that most likely represented returns to an operating room versus ancillary procedures (e.g., critical care procedures and monitoring, endoscopic procedures). We reviewed and grouped all remaining CCSR procedure categories into other clinically appropriate summary categories (Supplemental Table 1).

### Admission costs

The NIS provides total hospital charges and total costs of admission via the HCUP-supplied cost-to-charge ratio derived from Centers for Medicare and Medicaid Services cost reports. “Costs” defined in this manner refer to estimated healthcare system payouts based on nationally reported fee schedules. All costs were adjusted for inflation to 2019 dollars using the Consumer Price Index inflation calculator [16]. Among all rescued patients, admission cost percentiles were calculated; patients were dichotomized into those with “high-cost rescue” (costs at or above the 75th percentile [$67,077]) and those with “lower-cost rescue” (below the 75th percentile).

### Additional variables of interest

To determine the primary admitting diagnosis, the ICD-10-CM codes in the primary diagnosis field were categorized by CCSR and then organized by I.L. and K.S. into 6 clinically meaningful groups. The surgical approach used for the index surgery was determined as either minimally invasive or open by ICD-10-PCS code. Minimally invasive surgeries were further delineated as robotic- versus laparoscopic-assisted with the former designated by the presence of the ICD-10-PCS code beginning with 8E0Wxxx (“Robotic assisted procedure of the trunk region”) on the same day as the index surgery; all other minimally invasive cases were assumed to be laparoscopic.

Patient comorbidities were identified using ICD-10-CM codes in the secondary diagnosis fields. As the diagnosis codes in NIS are not tied to specific dates during admission or have indicators for present on admission, only comorbid conditions that were unlikely to have developed during admission were chosen (i.e., chronic conditions) (see Supplemental Table 2 for codes). Likewise, secondary diagnoses were examined to identify potential complications that occurred for patients in need of rescue. ICD-10-CM code descriptions and CCSR categories for secondary diagnoses were reviewed by I.L. and K.S. and grouped into categories of complication types (Supplemental Table 2).

### Statistical analysis

All analyses were appropriately weighted to produce national estimates. Demographic and clinical characteristics of rescued patients were summarized overall, by receipt of reoperation, and by high- versus lower-cost rescue. Distributions of categorical variables were compared across groups using Pearson's chi-square tests, while means were compared using *t*-tests and medians compared using weighted bi-variable quantile regression.

Total cost of admission was calculated for all rescued patients, as well as for those with and without reoperation; cost was calculated and graphed against length of stay to further describe the distribution and variability in costs among rescued patients.

Weighted, multivariable Poisson regression was used to estimate the risk of high-cost rescue by reoperation status. Potential confounders for adjustment were selected through a theory-driven approach with consideration for a parsimonious model if the effect estimate of interest was not substantially changed by the addition or removal of a given covariate. Only covariates that were reasonably believed to fully precede a reoperation were considered. The covariates used included age categories, sex, race/ethnicity, procedure approach, primary diagnosis, hospital bed size, and hospital teaching status. Effect modification was evaluated by stepwise testing for significance of covariate interactions with the exposure of interest.

### Sensitivity analysis

The effect of reoperation on cost group assignment was evaluated using both inverse probability weighting with regression adjustment (IPWRA) and propensity score matching with 1:1 nearest-neighbor matching. The IPWRA approach used the same covariates as the multivariable regression approach to model the outcome (high- versus low-cost rescue) and used a logistic model with the same covariates, as well as additional comorbidities, to predict reoperation status. The propensity score matching approach used the same covariates as the regression, as well as additional comorbidities, to model the propensity scores using a logit model. Standardized differences, balance plots, and tests for overidentification were used to evaluate covariate balance over the different treatment levels for both approaches. Time to reintervention as a covariate was modeled for less expensive versus more expensive rescued patients, and there was no difference.

All statistical analyses were performed using Stata 17.0 MP (StataCorp, College Station, TX). The reporting of findings is in accordance with guidelines established by the Strengthening the Reporting of Observational Studies in Epidemiology (STROBE) Statement.

## Results

We identified 448,590 elective colectomies and proctectomies in the NIS 2016–2019 data. Rescued patients composed 4.8 % (21,635) of the total sample. Table 1 provides preoperative characteristics of patients who were uneventfully discharged versus rescued. For rescued patients, results are further stratified by the less expensive group versus the most expensive patients as defined above. The most clinically relevant differences between the three groups were the proportion with underlying major comorbidities and the index resection procedural approach. Any major comorbidity was present in 40.1 % in uneventfully discharged versus 50.8 % in less expensive rescued patients versus 60.6 % in the most expensive rescued patients (*p* < 0.001). Most notably, congestive heart failure was closely correlated with being in the most expensive rescued subgroup (15.5 % versus 8.8 % in less expensive rescued versus 4.0 % in uneventfully discharged, *p* < 0.001). An open approach was used among 45.3 % of uneventfully discharged patients and >60 % in rescued groups (p < 0.001). There were no additional clinically relevant differences between the less expensive and most expensive subgroups (Supplemental Table 3).

**Table 1:** Demographic and clinical characteristics of patients hospitalized for elective colorectal resection in NIS 2016–2019, by admission outcome.

	Hospitalization course								*p*-Value
	Uneventful discharge		Rescued-less expensive		Rescued-most expensive		Total		
	N	%	N	%	N	%	N	%	
Number of admissions	426,955	95.2	16,230	3.6	5405	1.2	448,590	100.0	
Year of admission									
2016	104,840	24.6	4335	26.7	1370	25.3	110,545	24.6	0.199
2017	107,640	25.2	4115	25.4	1355	25.1	113,110	25.2	
2018	107,540	25.2	4025	24.8	1305	24.1	112,870	25.2	
2019	106,935	25.0	3755	23.1	1375	25.4	112,065	25.0	
Patient sex									
Female	227,580	53.3	7695	47.4	2485	46.0	237,760	53.0	<0.001
Male	199,375	46.7	8535	52.6	2920	54.0	210,830	47.0	
Patient age (continuous)									
Mean (SD)	62.2	13.9	62.8	14.7	63.5	13.4	62.2	12.7	0.001
Median (IQR)	63	53–72	64	54–73	65	55–73	63	53–72	<0.001
Patient age at admission									
<45	45,950	10.8	1825	11.2	475	8.8	48,250	10.8	<0.001
45–54	71,630	16.8	2405	14.8	795	14.7	74,830	16.7	
55–64	108,965	25.5	4025	24.8	1340	24.8	114,330	25.5	
65–74	118,195	27.7	4400	27.1	1685	31.2	124,280	27.7	
75+	82,215	19.3	3575	22.0	1110	20.5	86,900	19.4	
Patient race/ethnicity									
White	324,440	76.0	12,095	74.5	3800	70.3	340,335	75.9	<0.001
Black	36,660	8.6	1675	10.3	585	10.8	38,920	8.7	
Hispanic	30,840	7.2	1085	6.7	455	8.4	32,380	7.2	
Other	21,000	4.9	770	4.7	385	7.1	22,155	4.9	
Missing	14,015	3.3	605	3.7	180	3.3	14,800	3.3	
Primary admitting diagnosis									
Colorectal cancer	185,245	43.4	7175	44.2	2240	41.4	194,660	43.4	<0.001
Diverticular disease	95,555	22.4	2715	16.7	565	10.5	98,835	22.0	
Inflammatory bowel disease	21,670	5.1	1130	7.0	220	4.1	23,020	5.1	
Ostomy revision/closure	5555	1.3	385	2.4	160	3.0	6100	1.4	
Other neoplasia	81,555	19.1	2820	17.4	1350	25.0	85,725	19.1	
Other benign	37,375	8.8	2005	12.4	870	16.1	40,250	9.0	
Patient insurance									
Medicare	195,260	45.7	8020	49.4	2815	52.1	206,095	45.9	<0.001
Medicaid	29,525	6.9	1510	9.3	610	11.3	31,645	7.1	
Private	186,990	43.8	6040	37.2	1740	32.2	194,770	43.4	
Other	14,730	3.5	640	3.9	230	4.3	15,600	3.5	
Missing	450	0.1	20	0.1	10	0.2	480	0.1	
Patient zipcode-based income quartile									
First quartile	95,445	22.4	4285	26.4	1285	23.8	101,015	22.5	<0.001
Second quartile	108,000	25.3	4385	27.0	1395	25.8	113,780	25.4	
Third quartile	111,140	26.0	4025	24.8	1245	23.0	116,410	26.0	
Forth quartile	106,240	24.9	3275	20.2	1345	24.9	110,860	24.7	
Missing	6130	1.4	260	1.6	135	2.5	6525	1.5	
Any comorbidity	171,390	40.1	8245	50.8	3275	60.6	182,910	40.8	<0.001
Diabetes	81,505	19.1	3580	22.1	1195	22.1	86,280	19.2	<0.001
Coronary artery disease	47,485	11.1	2525	15.6	850	15.7	50,860	11.3	<0.001
Congestive heart failure	17,210	4.0	1430	8.8	840	15.5	19,480	4.3	<0.001
Atrial fibrillation	30,700	7.2	2250	13.9	1110	20.5	34,060	7.6	<0.001
Chronic kidney disease	11,610	2.7	910	5.6	345	6.4	12,865	2.9	<0.001
Chronic liver disease	3840	0.9	245	1.5	105	1.9	4190	0.9	<0.001
Chronic lung disease	63,560	14.9	3255	20.1	1205	22.3	68,020	15.2	<0.001
Resection procedure approach									
Open procedure	193,470	45.3	9790	60.3	3700	68.5	206,960	46.1	<0.001
Laparoscopic assisted	152,140	35.6	3970	24.5	890	16.5	157,000	35.0	
Robotic assisted	81,345	19.1	2470	15.2	815	15.1	84,630	18.9	
Hospital bed size									
Small	74,155	17.4	2385	14.7	695	12.9	77,235	17.2	<0.001
Medium	121,950	28.6	4725	29.1	1395	25.8	128,070	28.5	
Large	230,850	54.1	9120	56.2	3315	61.3	243,285	54.2	
Hospital location and teaching status									
Rural	29,970	7.0	1245	7.7	280	5.2	31,495	7.0	0.001
Urban non-teaching	79,280	18.6	2730	16.8	815	15.1	82,825	18.5	
Urban teaching	317,705	74.4	12,255	75.5	4310	79.7	334,270	74.5	
Hospital region									
Northeast	80,880	18.9	2930	18.1	970	17.9	84,780	18.9	<0.001
Midwest	109,635	25.7	4315	26.6	1140	21.1	115,090	25.7	
South	157,820	37.0	6375	39.3	1645	30.4	165,840	37.0	
West	78,620	18.4	2610	16.1	1650	30.5	82,880	18.5	

**Fig. 1:**
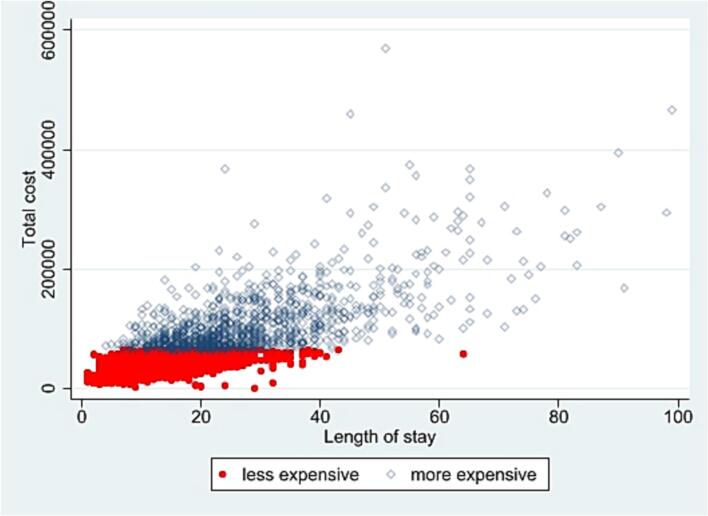
Comparison of length of stay relative to total inpatient costs (in U.S. dollars) for colorectal resection with in-hospital rescue in NIS 2016–2019. Patients denoted with open blue circles are the most expensive (75th percentile) by total inpatient cost, and the remainder are marked in red. The x-axis is length of stay, truncated to 100 days. The y-axis is total inpatient costs, truncated to $600,000. Truncation was performed for better visualization and only excluded 10 patients. (For interpretation of the references to colour in this figure legend, the reader is referred to the web version of this article.)

Postoperatively, the most expensive rescued patients had significant higher healthcare utilization (Table 2). Median lengths of stay were 26 days in the most expensive rescued versus 11 days in the less expensive rescued versus 4 in the uneventfully discharged (*p* < 0.001). They were disproportionately discharged to other than routine care (22.8 % in uneventfully discharged versus 84.4 %, *p* < 0.001). Median total inpatient costs were $95,926 in the most expensive rescued patients versus $34,811 in the less expensive rescued versus $16,751 in the uneventfully discharged (p < 0.001). The overall median cost for rescued patients was $42,295. Length of stay was associated with increased costs, but the variability dramatically increased with increased lengths of stay (Fig. 1). All of these differences were significant for three-way comparisons between all groups as well as two-way comparisons between rescued subgroups (Supplemental Table 4).

**Table 2:** Healthcare utilization of patients hospitalized for elective colorectal resection in NIS 2016–2019, by admission outcome.

	Hospitalization course								p-Value
	Uneventful discharge		Rescued-less expensive		Rescued-most expensive		Total		
	N	%	N	%	N	%	N	%	
Number of admissions	426,955	95.2	16,230	3.6	5405	1.2	448,590	100.0	
Discharge disposition									
Routine	329,725	77.2	7095	43.7	845	15.6	337,665	75.3	<0.001
Transfer-short-term hospital	955	0.2	210	1.3	130	2.4	1295	0.3	
Other transfer (eg SNF)	23,325	5.5	3330	20.5	2690	49.8	29,345	6.5	
Home health care	72,540	17.0	5555	34.2	1725	31.9	79,820	17.8	
Other/unknown	410	0.1	40	0.2	15	0.3	465	0.1	
LOS (continuous) (days)									
Mean (SD)	4.8	3.3	12.1	6.5	29.4	16.7	5.3	4.5	<0.001
Median (IQR)	4	3–6	11	7–16	26	19–35	4	3–6	0.031
LOS > 14 days	7420	1.7	5100	31.4	4765	88.2	17,285	3.9	<0.001
Total costs ($)									
Mean (SD)	19,706	11,778	36,171	14,351	117,991	73,344	21,486	16,509	<0.001
Median (IQR)	16,751	12,611-23,116	34,811	24,797-47,251	95,926	78,145-131,686	17,177	12,797-24,271	<0.001
Total costs/day^a^ ($)									
Mean (SD)	3740	1937	3097	1379	4273	1813	3723	1754	<0.001
Median (IQR)	3283	2452-4513	2818	2175-3657	3884	3041-5069	3269	2445-4487	0.005

a Denominator is LOS + 1 to account for same-day discharges (LOS = 0).

When comparing the secondary procedures between the less expensive and most expensive rescue groups (Table 3), the most expensive group had an increased proportion of patients undergoing a reoperation (73.4 % versus 53.0 %, *p* < 0.001). The most common procedures when returning to the operating room included diversion (19.1 %) of top ten most common), re-closure of the abdominal wall (15.1 %), and bowel resection (46.4 %) (Complete list, Supplemental Table 5). Among rescued patients, reoperation was associated with an increase in median total costs of $13,259 (*p* < 0.001). Complication-related secondary diagnoses were also more common in the most expensive rescued group with the greatest clinical differences being in the rate of localized infection (63.7 % versus 38.5 %, *p* < 0.001), sepsis (45.8 % versus 11.2 %, p < 0.001), and secondary end-organ injury (73.5 % versus 11.2 %, p < 0.001).

**Table 3:** Comparison of rescue for elective colorectal resection in NIS 2016–2019, by cost group.

	Cost groups for rescued patients						p-Value
	Less expensive		Most expensive		Total		
	N	%	N	%	N	%	
Number of admissions	16,230	75.0	5405	25.0	21,635	100.0	
Number of post-resection procedures							
1	10,100	62.2	1225	22.7	11,325	52.3	<0.001
2	3425	21.1	975	18.0	4400	20.3	
3	1525	9.4	860	15.9	2385	11.0	
4+	1180	7.3	2345	43.4	3525	16.3	
Categorized secondary procedures							
Reoperations	8595	53.0	3965	73.4	12,560	58.1	<0.001
Critical care procedures	5370	33.1	3125	57.8	8495	39.3	<0.001
Advanced endoscopic procedures	2835	17.5	995	18.4	3830	17.7	0.578
Secondary end-organ support	1905	11.7	595	11.0	2500	11.6	0.593
Supportive care procedures	155	1.0	105	1.9	260	1.2	0.032
Miscellaneous other procedures	150	0.9	40	0.7	190	0.9	0.608
Additional diagnoses during admission (categorized)							
Localized surgical infection	6255	38.5	3445	63.7	9700	44.8	<0.001
Intestinal obstruction and ileus	6525	40.2	2860	52.9	9385	43.4	<0.001
Postoperative hemorrhage	4765	29.4	2660	49.2	7425	34.3	<0.001
Nonspecified surgical complication	3925	24.2	2450	45.3	6375	29.5	<0.001
Secondary end-organ injury (kidney, lung, CNS, etc.)	5560	34.3	3975	73.5	9535	44.1	<0.001
Sepsis	1820	11.2	2475	45.8	4295	19.9	<0.001
Ostomy complications	630	3.9	325	6.0	955	4.4	0.015

When controlling for other factors with multivariable Poisson regression and stratification by congestive heart failure due to an interaction effect noted in early analyses, a reoperation was independently associated with high-cost rescue (RR with CHF = 3.29, 95%CI: 2.69–4.04; RR without CHF = 2.29, 95%CI: 1.97–2.67). This finding was robust to sensitivity analyses that evaluated the sequence of multiple rescue procedures as well regression with and without causal inference matching methods.

## Discussion

In this study, we used the weighted generalizable cohort of the National Inpatient Sample to evaluate predictors of total inpatient costs of rescue after elective colorectal resection. As expected, we found that rescue is associated with an increased median length of stay by over 7 days, triples the need for enhanced post-hospital care, and more than doubles the median cost of admission. Interestingly, we found that while comorbidities are associated with the occurrence rate of complications, most comorbidities are not substantial drivers for additional costs independent of the rescue event. Moreover, and unique to this study, we found that the subset of rescued patients with high inpatient costs are disproportionately higher. Rescued patients from the 75th percentile of total inpatient costs subgroup are associated with lengths of stay >5 times longer. This same subgroup incurs total inpatient costs almost 6 times higher than non-rescued patients. Although unable to be quantified in this data, a residual cost burden by proxy of their rescue was that only 5 % of uneventful discharges went to skilled nursing facilities versus 21 % of less expensive rescued and half of most expensive rescued patients. Most clinically relevant, reoperation appears to be a significant independent driver of higher cost rescue.

Rescue efforts after complications from surgery are an essential component of a functioning healthcare quality ecosystem [17], and efforts to maintain healthcare resilience should be promoted [5,18]. However, one criticism of the rescue-as-quality paradigm has been that a potential distortion of quality measurement induced by the use of a failure-to-rescue metric is that high complication rates with albeit low mortality may be overly-tolerated [19,20]. In addition, these interventions incur disproportionate greater use of healthcare resources, and the consequences of resource utilization after rescue is not well understood.

The existing literature has few studies that have examined the costs of rescue. Chen et al. reviewed Medicare payments following rescue scenarios in hepato-pancreato-bliary operations and found that rescue events contributed an additional $8840 to the index hospitalization [8,9]. Pradarelli et al. similarly examined 30-day Medicare payments and found that index colectomy complications in patients discharged alive led to $33,934 in additional 30-day costs [21]. Our own study findings align well with these findings with more routine rescue costs contributing $25,544 of additional inpatient hospital charges. Unique qualities of our approach include a definition of rescue that uses post-index surgery procedures rather than administratively reported complication diagnostic codes. We believe this approach more accurately ascertains patients with serious complications given the real-world vagaries of how diagnostic codes in the postoperative period are applied to surgical episodes of care. Specifically, coders are more likely to identify and bill post-index procedures performed than the low-impact postoperative complications codes [13,14].

### Implications for future work

Rescue events after colorectal surgery highlight important opportunities for and prioritization of quality improvement. Not all complications or adverse outcomes are created equal. For example, a well-recognized limitation of some quality reporting is that easily measured but minimally impactful outcomes such as *superficial* surgical site infections are not patient-centered and may be overemphasized [22]. Quantifying the healthcare economic impact of rescue helps better characterize resource utilization for adverse outcomes and where efforts for quality improvement should be directed.

Our findings highlight that from a cost-effectiveness perspective, reducing the risks of reoperation may need to be prioritized to reduce high-cost outliers of colorectal surgery. In the context of healthcare system decision-making, care interventions that increase the risk of reoperation may require a higher clinical benefit to offset the disproportionate system risks of a subsequent rescue-directed reoperation.

An example can be drawn from our group's prior work with venous thromboembolism (VTE) prophylaxis. Extended VTE prophylaxis has a small clinical risk of reoperation for bleeding and significant but small benefit of preventing late-occurring postoperative VTE [23,24]. Although further study is required, the risk of bleeding with extended VTE prophylaxis may disproportionately weigh against its use. Given that extended VTE prophylaxis increases the risk of reoperation for bleeding - historically thought to be a mere marginal risk for the patient and a system's healthcare utilization – this study's findings caution further consideration of a preventative practice with unintended cost consequences. In another example, the cost-effectiveness of regionalizing rectal cancer may also be further informed by these findings. If postoperative complications and rescue events are reduced with traveling to high volume centers as evidence suggests [25,26], the benefit of regionalizing care may be further leveraged because the reduction in healthcare utilization liability reduces the sequelae of high-cost rescue events.

Conversely, our findings may suggest that prevention efforts for complications that are readily treatable without returns to the operating room may not be as detrimental to healthcare system resilience and resource use. Superficial surgical site infections, arrhythmias, and other non-surgical complications may be clinically important to correct for the individual patient but have a dramatically lower impact on the healthcare systems in which they occur. Our findings would support perioperative preventative practices that target returns to the operating room (e.g., enhanced bleeding control, anastomotic leak prevention). For example, the most cost-effective complication prevention interventions are likely prophylactic use of hemostatic agents and adjunctive intra-operative steps for preventing anastomotic leak rather than superficial surgical site infection efforts or cardiopulmonary postoperative monitoring.

### Limitations

This study is not without limitations. All data analyzed derived from NIS, a nationally representative dataset with its underlying data originally used for administrative billing purposes. The data has routinely been reused for retrospective studies, but conclusions must acknowledge that the findings represent actual care rather than a randomized comparison between exposure groups. The dataset also does not link specific diagnosis codes with each procedure code to allow clinical interpretation of indications for operations. Use of the National Inpatient Sample is further limited by selection bias and specifically that something other than the need for a post-index surgical procedure is what led to patients' being heavy resource use inpatient admission. In addition, NIS is only able to capture hospital costs from an index admission. Patients discharged and readmitted who then required rescue events are missing from our reported findings; and care costs that remained disproportionately higher in the post-discharge setting are not captured. It is likely that the difference in resource use between our uneventfully discharged and rescued categories understate the total healthcare cost impact. These limitations should not impact our conclusions in a meaningful way.

## Conclusions

High-cost rescue after colorectal surgery is associated with disproportionately greater healthcare utilization in terms of length of stay, discharge disposition, and total inpatient costs. Reoperation following index resection appears to be an independent driver of high-cost rescue. For cost-conscious care, preemptive strategies that focus on reducing reoperation-related postoperative complications may require further prioritization.

## Funding/financial support

This study was performed without outside funding or financial support.

## Ethics approval

The following methodology was reviewed by the Yale Institutional Review Board and deemed exempt due to nonidentifiable data in a publicly available dataset.

## Meeting presentation

An earlier version of the analysis in this manuscript was presented at the American Society of Colon and Rectal Surgeons Annual Scientific Meeting in Seattle, Washington on June 2–6, 2023.

## CRediT authorship contribution statement

All authors of the manuscript contributed significantly to this manuscript according to the criteria set forth by the guidelines of the International Committee of Medical Journal Editors (ICMJE).

ILL – Study design, data interpretation manuscript drafting and revision.

MSM – Study design, data analysis, manuscript revision.

KS – Data interpretation, manuscript drafting.

JKC – Study design, data analysis, manuscript revision.

HJP – Study design, manuscript revision.

AKM – Study design, manuscript revision.

VR – Study design, manuscript revision.

ES – Study design, data analysis and interpretation, manuscript revision.

## Declaration of competing interest

All authors declare no conflicts of interest to the current work presented here.

## Appendix A Supplementary data

Supplementary tables Image 1
