# Supplementary material for: More problems, more money: Identifying and predicting high-cost rescue after colorectal surgery
Source: Surg Open Sci. 2023 Oct 28;16:148–54. doi: 10.1016/j.sopen.2023.10.007 (PMC10656212; doi:10.1016/j.sopen.2023.10.007)
Supplement: Supplementary file 1 — Supplementary tables [file mmc1.pdf]

SUPPLEMENTAL TABLES

Supplemental Table 1 – Procedural Categorization by CCS

	Included in procedure category if procedure falls into listed CCS category description
**Procedure category**	**CCS category description**
Advanced endoscopic procedures	Hepatobiliary and pancreatic drainage
	Esophagogastroduodenoscopy (EGD) with biopsy
	Upper GI therapeutic procedures, NEC (endoscopic)
	Endoscopic control of bleeding
	GI system endoscopy without biopsy (diagnostic)
	GI system endoscopic therapeutic procedures
	Pancreatic and proximal biliary dilation and stenting
	Colonoscopy and proctoscopy with biopsy
	Common bile duct sphincterotomy and stenting
	Biliary and pancreatic calculus removal
	Lower GI therapeutic procedures, NEC (excluding open and laparoscopic)
	Diagnostic ERCP with or without biopsy
Critical care procedures	Airway intubation
	Paracentesis
	Tracheostomy
	Chest tube placement and therapeutic thoracentesis
	Bronchoscopy (diagnostic)
	Cardiovascular device procedures, NEC
	Thoracentesis (diagnostic)
	Bronchoscopy (therapeutic)
	Respiratory system procedures, NEC
	Pacemaker and defibrillator procedures
	Laryngoscopy (diagnostic)
	Cerebrospinal fluid shunt procedures

	Lumbar puncture
	Pericardial procedures
	CNS procedures, NEC
	ENT procedures, NEC
	Dental procedures
	Trachelectomy
	Spinal epidural catheter placement
	Placement of tunneled or implantable portion of a vascular access device
	Mediastinal procedures, NEC
	ENT diagnostic procedures (non-endoscopic)
	ENT diagnostic endoscopy (excluding laryngoscopy)
	Bronchoscopic excision and fulguration
	Other GI system device procedures
Miscellaneous procedures	Kidney and other urinary tract biopsy (non-endoscopic)
	Nail procedures
	Circumcision
	Lymph node biopsy
	Bone marrow biopsy
	Subcutaneous contraceptive implant
	Endocrine system biopsy
	Endocrine procedures, NEC
Reoperation	Splenectomy
	Ileostomy and colostomy
	Small bowel resection
	Other peritoneal cavity procedures
	Colectomy
	GI system repair (excluding anorectal)
	GI system lysis of adhesions
	Gastrostomy
	Exploration of peritoneal cavity
	Abdominal wall repair (including hernia)

Abdominal wall procedures, NEC
Subcutaneous tissue and fascia excision
Subcutaneous tissue and fascia procedures, NEC
Omentectomy or peritoneum resection
Skin excision and debridement
Ligation and embolization of vessels
Anorectal repair (3rd and 4th degree obstetrical repairs and other)
Proctectomy or anal resection
GI system drainage (excluding paracentesis)
Cholecystectomy
Lower GI therapeutic procedures, NEC (open and laparoscopic)
Lung, pleura, or diaphragm resection (open and thoracoscopic)
Gastro-jejunal bypass (including bariatric)
Skin graft
Appendectomy
Peripheral arterial bypass procedures
Chest wall procedures, NEC
Gastrectomy
Open and thoracoscopic pleural drainage
Hepatobiliary resection and ablation
Urinary diversion with anastomosis or ostomy
Release of lung and pleura
Perineal skin repair (1st degree obstetrical and other)
Male perineum procedures
Subcutaneous tissue, fascia, and muscle biopsy
Skin biopsy and diagnostic drainage
Nephrectomy and ureterectomy
Duodenal resection
Urinary tract repair
Embolectomy, endarterectomy, and related vessel procedures (non-endovascular; excluding carotid)

Vessel repair and replacement
Lymph node dissection
Spleen procedures (excluding splenectomy)
Saphenous vein harvest and other therapeutic vessel removal
Pancreatectomy
Female genital tract repair (excluding vulva)
Lymph node excision (therapeutic)
Liver biopsy
Inguinal and femoral hernia repair
Hysterectomy
Incision and drainage of subcutaneous tissue and fascia
Female lower genital tract excision
Cystectomy (including fulguration) and urethrectomy
Salpingectomy
Control of bleeding (non-endoscopic)
Incision and drainage of skin
Lung, pleura, or diaphragm biopsy (non-endoscopic)
Skin laceration repair (excluding perineum)
Prostatectomy
Prostate and seminal vesicle procedures (excluding prostatectomy)
Perineal muscle laceration repair (2nd degree obstetrical and other)
Female upper genital tract excision
Oophorectomy
Esophagectomy
Diaphragmatic hernia repair
Upper GI therapeutic procedures, NEC (open and laparoscopic)
Retroperitoneal procedures, NEC
GI system biopsy (non-endoscopic)
Hepatobiliary and pancreatic procedures, NEC
Urinary system procedures, NEC
Female reproductive system procedures, NEC

	Skin and breast procedures, NEC
Supportive care procedures	Inferior vena cava (IVC) filter procedures
Secondary end organ recovery procedures	Musculoskeletal procedures, NEC
	Muscle, tendon, bursa, and ligament excision
	Musculoskeletal device procedures, NEC
	Incision and drainage of musculoskeletal tissue and joints
	Bone excision
	Artery, vein, and great vessel procedures, NEC
	Hip arthroplasty
	Percutaneous coronary interventions (PCI)
	Angioplasty and related vessel procedures (endovascular; excluding carotid)
	Removal of calculi and other matter from urinary tract
	Bone and joint biopsy
	Nephrostomy and ureterostomy procedures (including stents)
	Cystoscopy and ureteroscopy (including biopsy)
	Ureter and other urinary tract dilation
	Closed reduction of bones and joints
	Peripheral arteriovenous fistula and shunt procedures
	Above knee and other proximal lower extremity amputation
	Below knee amputation
	Meninges repair
	Arthroplasty of other joint (excluding knee and hip)
	ENT repair
	CNS excision procedures
	Aneurysm repair procedures
	Arthrocentesis
	Joint tissue excision (excluding discectomy)
	Intracranial epidural and subdural space drainage
	Vertebral discectomy
	Ventriculostomy [percutaneous approach]

Toe and mid foot amputation
Tendon, muscle, bursa, and ligament repair (excluding perineal)
Coronary artery bypass grafts (CABG)
Bone fixation (excluding extremities)
Heart conduction mechanism procedures
Spine fusion
Heart assist device procedures

Supplemental Table 2 – Complications and Comorbidities

	Included in complication category if diagnosis code falls into any of the below (either has the specified CCS code description, category description, or is one of a handful of specified codes)		
**Complication category**	**CCS code description**	**CCS category description**	**Dx code**
Intestinal obstruction and ileus		Intestinal obstruction and ileus	K913
		Nausea and vomiting	K910
Postoperative hemorrhage	Hemorrhage due to internal prosthetic devices, implants and grafts, not elsewhere classified, initial encounter	Acute posthemorrhagic anemia	
	Postprocedural hemorrhage of a digestive system organ or structure following a digestive system procedure		
	Postprocedural hematoma of a digestive system organ or structure following a digestive system procedure		
	Intraoperative hemorrhage and hematoma of a digestive system organ or structure complicating a digestive system procedure		
	Postprocedural hemorrhage of a digestive system organ or structure following other procedure		
	Postprocedural hematoma of a digestive system organ or structure following other procedure		
	Intraoperative hemorrhage and hematoma of a digestive system organ or structure complicating other procedure		
Ostomy complications	Other mechanical complication of other gastrointestinal prosthetic devices, implants and grafts, initial encounter		
	Displacement of other gastrointestinal prosthetic devices, implants and grafts, initial encounter		
	Displacement of other specified internal prosthetic devices, implants and grafts, initial encounter		
	Other complications of colostomy		
	Other complications of enterostomy		
	Enterostomy malfunction		
	Colostomy malfunction		

	Gastrostomy malfunction	
	Enterostomy hemorrhage	
	Enterostomy infection	
	Colostomy infection	
	Colostomy hemorrhage	
	Enterostomy complication, unspecified	
	Other complications of gastrostomy	
Localized surgical infection	Accidental puncture and laceration of a digestive system organ or structure during a digestive system procedure	Abdominal hernia
	Accidental puncture and laceration of a digestive system organ or structure during other procedure	Gastrointestinal and biliary perforation
	Infection following a procedure, initial encounter	Peritonitis and intra-abdominal abscess
	Disruption of wound, unspecified, initial encounter	Skin and subcutaneous tissue infections
	Infection following a procedure, organ and space surgical site, initial encounter	
	Infection following a procedure, superficial incisional surgical site, initial encounter	
	Infection following a procedure, other surgical site, initial encounter	
	Infection following a procedure, unspecified, initial encounter	
	Persistent postprocedural fistula, initial encounter	
	Infection following a procedure, deep incisional surgical site, initial encounter	
	Postprocedural seroma of a digestive system organ or structure following a digestive system procedure	
	Postprocedural seroma of a digestive system organ or structure following other procedure	
	Infection due to gastric band procedure	
	Other complications of foreign body accidentally left in body following surgical operation, initial encounter	

	Aseptic peritonitis due to foreign substance accidentally left during a procedure, initial encounter		
	Unspecified complication of foreign body accidentally left in body following removal of catheter or packing, initial encounter		
	Unspecified complication of foreign body accidentally left in body following surgical operation, initial encounter		
Postoperative malabsorption	Postsurgical malabsorption, not elsewhere classified		
Sepsis		Septicemia	T8112XA
			T8144XA
Secondary end-organ injury (kidney, lung, CNS, etc.)	Emphysema (subcutaneous) resulting from a procedure, initial encounter	Shock	T8110XA
	Displacement of cranial or spinal infusion catheter, initial encounter	Postprocedural or postoperative respiratory system complication	T8111XA
	Anaphylactic reaction due to adverse effect of correct drug or medicament properly administered, initial encounter	Pleurisy, pleural effusion and pulmonary collapse	T8119XA
	Leakage of cranial or spinal infusion catheter, initial encounter	Acute and unspecified renal failure	
	Breakdown (mechanical) of muscle and tendon graft, initial encounter		
	Postprocedural hepatic failure		
Unspecific surgical complication	Disruption of internal operation (surgical) wound, not elsewhere classified, initial encounter		
	Disruption of external operation (surgical) wound, not elsewhere classified, initial encounter		
	Complication of surgical and medical care, unspecified, initial encounter		
	Other complications of procedures, not elsewhere classified, initial encounter		
	Bloodstream infection due to central venous catheter, initial encounter		
	Infection and inflammatory reaction due to other internal prosthetic devices, implants and grafts, initial encounter		

Leakage of other specified internal prosthetic devices, implants and grafts, initial encounter

Complication of other artery following a procedure, not elsewhere classified, initial encounter

Complication of vein following a procedure, not elsewhere classified, initial encounter

Vascular complications following infusion, transfusion and therapeutic injection, initial encounter

Other complications of anesthesia, initial encounter

Other complications following infusion, transfusion and therapeutic injection, initial encounter

Other specified complication of other internal prosthetic devices, implants and grafts, initial encounter

Unspecified infection due to central venous catheter, initial encounter

Other specified complication of internal prosthetic devices, implants and grafts, not elsewhere classified, initial encounter

Breakdown (mechanical) of other specified internal prosthetic devices, implants and grafts, initial encounter

Other mechanical complication of other specified internal prosthetic devices, implants and grafts, initial encounter

Extravasation of other vesicant agent, initial encounter

Breakdown (mechanical) of other nervous system device, implant or graft, initial encounter

Pain due to other internal prosthetic devices, implants and grafts, initial encounter

Thrombosis due to other internal prosthetic devices, implants and grafts, initial encounter

Infection following other infusion, transfusion and therapeutic injection, initial encounter

Unspecified complication of internal prosthetic device, implant and graft, initial encounter

	Infection and inflammatory reaction due to ventricular intracranial (communicating) shunt, initial encounter
	Other specified complication of nervous system prosthetic devices, implants and grafts, initial encounter
	Other postprocedural complications and disorders of digestive system
	Other complications of intestinal pouch
	Unspecified transfusion reaction, initial encounter
	Failed or difficult intubation, initial encounter
	Local infection due to central venous catheter, initial encounter
	Other specified complications of surgical and medical care, not elsewhere classified, initial encounter
	Other intraoperative complications of digestive system
	Pouchitis
	Postgastric surgery syndromes

Comorbidity	Diagnosis code	Code description
Diabetes	E10	Type 1 diabetes mellitus
	E11	Type 2 diabetes mellitus
Congestive heart failure	I11.0	Hypertensive heart disease with heart failure
	I13.0	Hypertensive heart and chronic kidney disease with heart failure and stage 1 through stage 4 chronic kidney disease, or unspecified chronic kidney disease
	I13.2	Hypertensive heart and chronic kidney disease with heart failure and with stage 5 chronic kidney disease, or end stage renal disease
	I50	Heart failure
Chronic kidney disease	I12.0	Hypertensive chronic kidney disease with stage 5 chronic kidney disease or end stage renal disease
	I13.11	Hypertensive heart and chronic kidney disease without heart failure, with stage 5 chronic kidney disease, or end stage renal disease
	I13.2	Hypertensive heart and chronic kidney disease with heart failure and with stage 5 chronic kidney disease, or end stage renal disease
	N18.4	Chronic kidney disease, stage 4 (severe)

	N18.5	Chronic kidney disease, stage 5
	N18.6	End stage renal disease
	N18.9	Chronic kidney disease, unspecified
	Z49	Encounter for care involving renal dialysis
	Z91.15	Patient's noncompliance with renal dialysis
	Z99.2	Dependence on renal dialysis
Coronary artery disease	I25	Chronic ischemic heart disease
Atrial fibrillation	I48	Atrial fibrillation and flutter
Chronic lung disease	J41	Simple and mucopurulent chronic bronchitis
	J42	Unspecified chronic bronchitis
	J43	Emphysema
	J44	Other chronic obstructive pulmonary disease
	J45	Asthma
	J47	Bronchiectasis
Chronic liver disease	K70	Alcoholic liver disease
	K74	Fibrosis and cirrhosis of liver
	K76	Other diseases of liver

Supplemental Table 3

Demographic and clinical characteristics of patients hospitalized for elective colorectal resection in NIS 2016-2019, by cost groups among rescued patients

	Cost groups for rescued patients						
	Less expensive		Most expensive		Total		
	N	%	N	%	N	%	p-value
**Number of admissions**	16,230	75.0	5,405	25.0	21,635	100.0	
**Year of admission**							
2016	4,335	26.7	1,370	25.3	5,705	26.4	0.692
2017	4,115	25.4	1,355	25.1	5,470	25.3	
2018	4,025	24.8	1,305	24.1	5,330	24.6	
2019	3,755	23.1	1,375	25.4	5,130	23.7	
**Patient sex**							
Female	7,695	47.4	2,485	46.0	10,180	47.1	0.498
Male	8,535	52.6	2,920	54.0	11,455	52.9	
**Patient age (continuous)**							
Mean (SD)	62.8	14.7	63.5	13.4	63.0	13.1	0.235
Median (IQR)	64	54-73	65	55-73	64	54-73	0.090
**Patient age at admission**							
<45	1,825	11.2	475	8.8	2,300	10.6	0.129
45-54	2,405	14.8	795	14.7	3,200	14.8	
55-64	4,025	24.8	1,340	24.8	5,365	24.8	
65-74	4,400	27.1	1,685	31.2	6,085	28.1	
75+	3,575	22.0	1,110	20.5	4,685	21.7	
**Patient race/ethnicity**							
White	12,095	74.5	3,800	70.3	15,895	73.5	0.048
Black	1,675	10.3	585	10.8	2,260	10.4	
Hispanic	1,085	6.7	455	8.4	1,540	7.1	
Other	770	4.7	385	7.1	1,155	5.3	

Missing	605	3.7	180	3.3	785	3.6	
**Primary admitting diagnosis**							
Colorectal cancer	7,175	44.2	2,240	41.4	9,415	43.5	0.000
Diverticular disease	2,715	16.7	565	10.5	3,280	15.2	
Inflammatory bowel disease	1,130	7.0	220	4.1	1,350	6.2	
Ostomy revision/closure	385	2.4	160	3.0	545	2.5	
Other neoplasia	2,820	17.4	1,350	25.0	4,170	19.3	
Other benign	2,005	12.4	870	16.1	2,875	13.3	
**Patient insurance**							
Medicare	8,020	49.4	2,815	52.1	10,835	50.1	0.105
Medicaid	1,510	9.3	610	11.3	2,120	9.8	
Private	6,040	37.2	1,740	32.2	7,780	36.0	
Other	640	3.9	230	4.3	870	4.0	
Missing	20	0.1	10	0.2	30	0.1	
**Patient zipcode-based income quartile**							
First quartile	4,285	26.4	1,285	23.8	5,570	25.7	0.030
Second quartile	4,385	27.0	1,395	25.8	5,780	26.7	
Third quartile	4,025	24.8	1,245	23.0	5,270	24.4	
Forth quartile	3,275	20.2	1,345	24.9	4,620	21.4	
Missing	260	1.6	135	2.5	395	1.8	
**Any comorbidity**	8,245	50.8	3,275	60.6	11,520	53.2	0.000
Diabetes	3,580	22.1	1,195	22.1	4,775	22.1	0.976
Coronary artery disease	2,525	15.6	850	15.7	3,375	15.6	0.911
Congestive heart failure	1,430	8.8	840	15.5	2,270	10.5	0.000
Atrial fibrillation	2,250	13.9	1,110	20.5	3,360	15.5	0.000
Chronic kidney disease	910	5.6	345	6.4	1,255	5.8	0.432
Chronic liver disease	245	1.5	105	1.9	350	1.6	0.400
Chronic lung disease	3,255	20.1	1,205	22.3	4,460	20.6	0.191
**Resection procedure approach**							
Open procedure	9,790	60.3	3,700	68.5	13,490	62.4	
Laparoscopic assisted	3,970	24.5	890	16.5	4,860	22.5	0.000

Robotic assisted	2,470	15.2	815	15.1	3,285	15.2	
**Hospital bed size**							
Small	2,385	14.7	695	12.9	3,080	14.2	0.057
Medium	4,725	29.1	1,395	25.8	6,120	28.3	
Large	9,120	56.2	3,315	61.3	12,435	57.5	
**Hospital location and teaching status**							
Rural	1,245	7.7	280	5.2	1,525	7.0	0.020
Urban non-teaching	2,730	16.8	815	15.1	3,545	16.4	
Urban teaching	12,255	75.5	4,310	79.7	16,565	76.6	

Supplemental Table 4
Healthcare utilization of patients hospitalized for elective colorectal resection in in NIS 2016-2019, by cost groups for rescued patients

	Cost groups for rescued patients						
	Less expensive		Most expensive		Total		
	N	%	N	%	N	%	p-value
**Number of admissions**	16,230	75.0	5,405	25.0	21,635	100.0	
**Discharge disposition**							
Routine	7,095	43.7	845	15.6	7,940	36.7	<0.001
Transfer-short-term hospital	210	1.3	130	2.4	340	1.6	
Other transfer (eg SNF)	3,330	20.5	2,690	49.8	6,020	27.8	
Home health care	5,555	34.2	1,725	31.9	7,280	33.6	
Other/Unknown	40	0.2	15	0.3	55	0.3	
**LOS (continuous) (days)**							
Mean (SD)	12.1	6.5	29.4	16.7	16.4	11.4	<0.001
Median (IQR)	11	7-16	26	19-35	14	8-21	<0.001
**LOS >14 days**	5,100	31.4	4,765	88.2	9,865	45.6	<0.001
**Total costs ($)**							
Mean (SD)	36,171	14,351	117,991	73,344	56,612	47,878	<0.001
Median (IQR)	34,811	24,797-47,251	95,926	78,145-131,686	42,295	27,959-67,077	<0.001
**Total costs/day* ($)**							
Mean (SD)	3,097	1,379	4,273	1,813	3,390	1,445	<0.001
Median (IQR)	2,818	2,175-3,657	3,884	3,041-5,069	3,049	2,312-4,005	<0.001

Supplemental Table 5

Count of patients with specific reoperation procedure codes, unweighted (limited to those with >10 patients)

ICD-10-PCS code	Procedure code description	Patient count
0D1B0Z4	Bypass Ileum to Cutaneous, Open Approach	389
0DBB0ZZ	Excision of Ileum, Open Approach	188
0WQF0ZZ	Repair Abdominal Wall, Open Approach	151
0DBL0ZZ	Excision of Transverse Colon, Open Approach	99
0DB80ZZ	Excision of Small Intestine, Open Approach	98
0DN80ZZ	Release Small Intestine, Open Approach	98
0JQ80ZZ	Repair Abdomen Subcutaneous Tissue and Fascia, Open Approach	90
0D1L0Z4	Bypass Transverse Colon to Cutaneous, Open Approach	82
0DH63UZ	Insertion of Feeding Device into Stomach, Percutaneous Approach	75
0DQ80ZZ	Repair Small Intestine, Open Approach	73
0D1M0Z4	Bypass Descending Colon to Cutaneous, Open Approach	71
0W9G0ZZ	Drainage of Peritoneal Cavity, Open Approach	68
0W9F30Z	Drainage of Abdominal Wall with Drainage Device, Percutaneous Approach	65
0WJP0ZZ	Inspection of Gastrointestinal Tract, Open Approach	61
0D1B4Z4	Bypass Ileum to Cutaneous, Percutaneous Endoscopic Approach	58
0JB80ZZ	Excision of Abdomen Subcutaneous Tissue and Fascia, Open Approach	57
0WUF0JZ	Supplement Abdominal Wall with Synthetic Substitute, Open Approach	56
0WJG4ZZ	Inspection of Peritoneal Cavity, Percutaneous Endoscopic Approach	56
0WJG0ZZ	Inspection of Peritoneal Cavity, Open Approach	52
0W9G00Z	Drainage of Peritoneal Cavity with Drainage Device, Open Approach	51
0DQB0ZZ	Repair Ileum, Open Approach	44
0WQFXZ2	Repair Abdominal Wall, Stoma, External Approach	44
0WCG0ZZ	Extirpation of Matter from Peritoneal Cavity, Open Approach	39
0DBM0ZZ	Excision of Descending Colon, Open Approach	38

0DQE0ZZ	Repair Large Intestine, Open Approach	38
0DBU0ZZ	Excision of Omentum, Open Approach	37
0D1B0ZL	Bypass Ileum to Transverse Colon, Open Approach	36
0WJP4ZZ	Inspection of Gastrointestinal Tract, Percutaneous Endoscopic Approach	35
0DBE0ZZ	Excision of Large Intestine, Open Approach	35
0D1N0Z4	Bypass Sigmoid Colon to Cutaneous, Open Approach	35
0DNW0ZZ	Release Peritoneum, Open Approach	35
0DN84ZZ	Release Small Intestine, Percutaneous Endoscopic Approach	35
0DBN0ZZ	Excision of Sigmoid Colon, Open Approach	34
0W9J0ZZ	Drainage of Pelvic Cavity, Open Approach	30
0DJD4ZZ	Inspection of Lower Intestinal Tract, Percutaneous Endoscopic Approach	29
0WQFXZZ	Repair Abdominal Wall, External Approach	28
0W9F0ZZ	Drainage of Abdominal Wall, Open Approach	25
0W2GX0Z	Change Drainage Device in Peritoneal Cavity, External Approach	24
0W9J00Z	Drainage of Pelvic Cavity with Drainage Device, Open Approach	23
0W9H30Z	Drainage of Retroperitoneum with Drainage Device, Percutaneous Approach	23
0W9F3ZZ	Drainage of Abdominal Wall, Percutaneous Approach	22
0J9C30Z	Drainage of Pelvic Region Subcutaneous Tissue and Fascia with Drainage Device, Percutaneous Approach	22
0DBS0ZZ	Excision of Greater Omentum, Open Approach	21
0HQ7XZZ	Repair Abdomen Skin, External Approach	20
0DQP0ZZ	Repair Rectum, Open Approach	20
0DBA0ZZ	Excision of Jejunum, Open Approach	19
0DTF0ZZ	Resection of Right Large Intestine, Open Approach	19
0DTL0ZZ	Resection of Transverse Colon, Open Approach	19
0DH60UZ	Insertion of Feeding Device into Stomach, Open Approach	19
0W9G40Z	Drainage of Peritoneal Cavity with Drainage Device, Percutaneous Endoscopic Approach	18
0DQL0ZZ	Repair Transverse Colon, Open Approach	18
0JBB0ZZ	Excision of Perineum Subcutaneous Tissue and Fascia, Open Approach	18
0DNB0ZZ	Release Ileum, Open Approach	18

0H97XZZ	Drainage of Abdomen Skin, External Approach	17
0WQF4ZZ	Repair Abdominal Wall, Percutaneous Endoscopic Approach	17
0DBP0ZZ	Excision of Rectum, Open Approach	16
0FT40ZZ	Resection of Gallbladder, Open Approach	16
0J980ZZ	Drainage of Abdomen Subcutaneous Tissue and Fascia, Open Approach	16
0W3G0ZZ	Control Bleeding in Peritoneal Cavity, Open Approach	16
0JC80ZZ	Extirpation of Matter from Abdomen Subcutaneous Tissue and Fascia, Open Approach	15
0DT80ZZ	Resection of Small Intestine, Open Approach	15
0DQM0ZZ	Repair Descending Colon, Open Approach	15
0JD80ZZ	Extraction of Abdomen Subcutaneous Tissue and Fascia, Open Approach	14
0TQB0ZZ	Repair Bladder, Open Approach	14
0W2FX0Z	Change Drainage Device in Abdominal Wall, External Approach	14
0DTG0ZZ	Resection of Left Large Intestine, Open Approach	14
0DTJ0ZZ	Resection of Appendix, Open Approach	14
0W2JX0Z	Change Drainage Device in Pelvic Cavity, External Approach	13
0W3P0ZZ	Control Bleeding in Gastrointestinal Tract, Open Approach	13
0DNU0ZZ	Release Omentum, Open Approach	13
0DTM0ZZ	Resection of Descending Colon, Open Approach	13
0W9G4ZZ	Drainage of Peritoneal Cavity, Percutaneous Endoscopic Approach	13
0DQA0ZZ	Repair Jejunum, Open Approach	12
0WUF07Z	Supplement Abdominal Wall with Autologous Tissue Substitute, Open Approach	12
0DTN0ZZ	Resection of Sigmoid Colon, Open Approach	12
0D1N4Z4	Bypass Sigmoid Colon to Cutaneous, Percutaneous Endoscopic Approach	12
0W9J40Z	Drainage of Pelvic Cavity with Drainage Device, Percutaneous Endoscopic Approach	12
0DBK0ZZ	Excision of Ascending Colon, Open Approach	11
0DNB4ZZ	Release Ileum, Percutaneous Endoscopic Approach	11
0WPF0JZ	Removal of Synthetic Substitute from Abdominal Wall, Open Approach	11
0DBB4ZZ	Excision of Ileum, Percutaneous Endoscopic Approach	11
0DQV0ZZ	Repair Mesentery, Open Approach	11

0DQN0ZZ	Repair Sigmoid Colon, Open Approach	11
0DNE0ZZ	Release Large Intestine, Open Approach	11
0WUF0KZ	Supplement Abdominal Wall with Nonautologous Tissue Substitute, Open Approach	11
